# Comparison of neurodevelopmental outcomes of extremely preterm infants undergoing trans-catheter closure of the patent ductus arteriosus compared to surgical ligation

**DOI:** 10.1038/s41372-025-02417-8

**Published:** 2025-09-23

**Authors:** D. C. Kaluarachchi, V. Y. Chock, B. T. Do, M. A. Rysavy, M. N. Sankar, M. M. Laughon, C. H. Backes, T. T. Colaizy, E. F. Bell, P. J. McNamara, S. R. Hintz, G. Natarajan

**Affiliations:** 1 Department of Pediatrics, University of Wisconsin-Madison, Madison, WI USA; 2 Stanford University, Palo Alto, CA USA; 3 Statistics Division RTI International, Research Triangle Park, Chapel Hill, USA; 4 McGovern Medical School, UTHealth Houston, Houston, TX USA; 5 University of North Carolina, Chapel Hill, NC USA; 6 Ohio State University and Nationwide Children’s Hospital, Columbus, OH USA; 7 Department of Pediatrics, University of Iowa, Iowa City, IA USA; 8 Department of Pediatrics, Wayne State University, Detroit, MI USA

**Keywords:** Cardiovascular diseases, Paediatrics, Outcomes research

## Abstract

**Background:**

There is a paucity of data on neurodevelopmental outcomes in preterm infants who undergo transcatheter patent ductus arteriosus (PDA) closure (TCPC).

**Objective:**

To evaluate neurodevelopmental impairment (NDI) or death at 2 years among preterm infants treated with TCPC compared to surgical ligation.

**Methods:**

Retrospective cohort study of infants born at <27 weeks’ gestation at NICHD NRN sites. Comparisons were made between infants who underwent TCPC and PDA ligation.

**Results:**

TCPC and surgical ligation were performed on 99 and 279 infants, respectively. Death or severe NDI occurred in 49% of infants with TCPC and 40% with surgical ligation. There was no difference in odds of death or severe NDI between the two groups [aOR 1.12 95% CI: 0.55–2.26)].

**Conclusion:**

TCPC had similar odds of death or severe NDI compared to surgical ligation. These findings need to be evaluated in large prospective studies as the management practice around the TCPC evolves.

**Clinical trial registration:**

ClinicalTrials.gov ID: Generic Database: NCT00063063.

## Introduction

Transcatheter patent ductus arteriosus (PDA) closure (TCPC) has become an increasingly common treatment for preterm infants [1–3]. Between 2016 and 2021, there has been a 4-fold increase in the number of patients undergoing TCPC [1]. In 2021, there were more than twice as many episodes of TCPC as surgical ligation [1]. This trend has continued, despite calls for caution by experts in the field due to limited evidence [4, 5]. Data from several observational studies showed superior short-term outcomes following TCPC compared to surgical ligation [6–9]. In contrast, multicenter data from the National Institute of Child Health and Human Development (NICHD) Neonatal Research Network (NRN) showed similar respiratory outcomes in infants treated with TCPC and surgical ligation [10].

Previous data have shown an association between surgical ligation and neurodevelopmental impairment (NDI); however, some of these studies did not consider pre-ligation morbidities, the procedure itself, or post ligation cardiac syndrome [11–14]. A study conducted by Weisz and colleagues, reported no difference in the odds of death or NDI between surgical ligation or medical treatment after adjusting for differences in perinatal characteristics and pre-ligation morbidities [15].

There is a paucity of data on neurodevelopmental outcomes in extremely preterm infants who undergo TCPC. Data from two small observational studies that included a total of 25 subjects undergoing TCPC found no difference in neurodevelopmental outcomes in patients undergoing TCPC compared to surgical ligation [16, 17]. No large multicenter studies has evaluated neurodevelopmental outcomes in preterm infants undergoing TCPC.

The objective of the current study was to evaluate death or neurodevelopmental outcomes of extremely preterm infants undergoing TCPC compared to surgical ligation.

## Methods

We performed a retrospective cohort study of preterm infants born at 22 0/7 through 26 6/7 weeks’ gestation at centers participating in the NICHD NRN between 1/1/2016 and 12/31/2019. Infants who died before 12 postnatal hours, were born outside of NRN hospitals or had major congenital anomalies were excluded.

We used prospectively collected data from the NRN Generic Database and linked Follow-up Database. Information on PDA diagnosis and treatment was prospectively entered by trained data abstractors. PDA diagnosis (“yes or no”) was defined as documentation of clinical or echocardiographic evidence of left-to-right PDA physiology determined by the clinical team in routine care. Medical PDA treatment was defined as any medication used specifically to induce PDA closure (indomethacin, ibuprofen, or acetaminophen) regardless of timing, duration, or dose. Procedural PDA closure included transcatheter and transthoracic surgical closure. Neonatal characteristics (e.g., gestational age at birth, birth weight, sex) were also collected.

The follow-up visit at 22–26 months’ corrected age included standardized physical and neurologic examination and the Bayley Scales of Infant and Toddler Development, Third Edition (Bayley-III) [18], both of which were administered by certified examiners who completed annual training to ensure interrater reliability [19]. Bayley-III cognitive, language, and motor composite scores are normalized to a mean of 100 (standard deviation 15). Severity of motor impairment was determined by the Gross Motor Function Classification system (GMFCS) of Palisano et al. [20]. Mild cerebral palsy (CP) as defined as GMFCS level 1, moderate as GMFCS level 2 or 3, and severe as GMFCS level 4 or 5.

### Outcomes

The primary outcome was severe NDI or death before developmental assessment. Severe NDI was defined as any of the following: motor and/or cognitive composite score <70 on Bayley-III, gross motor impairment with GMFCS level 4 or 5, bilateral blindness or deafness. Secondary outcomes were death, severe NDI, moderate to severe NDI (any of Bayley-III cognitive or motor composite score < 85, GMFCS > 2, bilateral blindness or deafness), any CP, severity of CP, Bayley-III cognitive, language and motor composite scores, Bayley-III cognitive, language, and motor composite score <70 and head growth (Z score at follow-up).

### Statistical analyses

Descriptive statistics included median (IQR) for continuous variables and frequencies and proportions for categorical variables. Unadjusted comparisons of baseline characteristics and neonatal morbidities among infants grouped by treatment received (TCPC vs surgery) were performed using chi-squared or Fisher’s exact tests for categorical variables and analysis of variance or Kruskal-Wallis tests for continuous variables.

To assess the relationship between PDA procedural treatment strategy and outcomes, we performed a multivariable logistic regression analysis with the outcome of severe NDI or death adjusting for the center, birth year, gestational age (in weeks), birth weight (in grams) and postnatal age (in days) at PDA intervention.

Statistical significance was defined as a two-sided *p*-value < 0.05 with no adjustment for multiple testing. All analyses were done using SAS (v. 9.4).

### Ethics approval and consent to participate

Participating centers received local institutional review board (IRB) approval for data collection. Per individual IRB requirements, data were collected under a waiver of consent or after informed consent was obtained from parents or legal guardians. All study procedures were performed in accordance with the relevant guidelines and regulations.

## Results

**Fig. 1:**
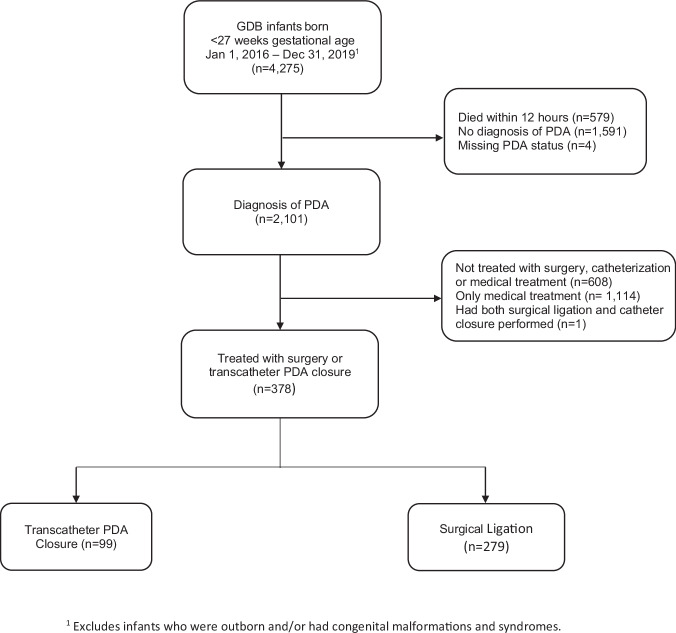
CONSORT Diagram. ^1^Excludes infants who were outborn and/or had congenital malformations and syndromes.

The study cohort included 378 infants who received procedural closure of their PDA. TCPC and surgical ligation were performed on 99 and 279 infants, respectively (Fig. 1). Neurodevelopmental follow up data were available for 78 and 197 infants respectively.

Characteristics of infants in these groups are presented in Table 1. Median gestational age (25.0 vs 24.7 weeks) and birth weight (690 vs 680 g) were not significantly different between the TCPC and surgical ligation groups. The median age at TCPC was almost twofold greater than the age at ligation (62 vs. 32 days, *p* < 0.01). There were more females in the TCPC group compared to the surgical ligation group. Median postmenstrual age was 33.9 weeks for TCPC and 29.1 for surgical ligation. The incidence of intraventricular hemorrhage was similar between the groups, but there was more periventricular leukomalacia in the TCPC group (16% vs 8%, *p* = 0.03). Data on PDA treatment and inpatient morbidities are presented in Table 2.

**Table 1:** Perinatal Characteristics.

Characteristics	*n*	Catheter closure *n* = 99	n	Surgical ligation *n* = 279	*P*-value^a^
Birth weight, g, Median (IQR)	99	690 (600–790)	279	680 (580–790)	0.32
Gestational Age, weeks, Median (IQR)	99	25.0 (24.0–25.9)	279	24.7 (23.9–25.4)	0.07
Male, *n* (%)	99	36 (36)	279	136 (49)	0.03
APGAR at 5 min < 5, *n* (%)	99	25 (25)	278	60 (22)	0.45
Small for gestational age, *n* (%)	99	6 (6)	279	16 (6)	0.91
Maternal age, years, Median (IQR)	99	29 (24–31)	279	29 (24–33)	0.48
Single marital status, *n* (%)	99	54 (55)	277	146 (53)	0.75
Highest level of maternal education, *n* (%)					
<High school degree	84	13 (15)	251	36 (14)	0.94
High school degree	84	26 (31)	251	87 (35)	
College degree	84	20 (24)	251	58 (23)	
Graduate degree	84	25 (30)	251	70 (28)	
Magnesium sulfate, n (%)	99	82 (83)	279	234 (84)	0.81
Public medical insurance, n (%)	99	50 (51)	279	158 (57)	0.29
Prenatal care, *n* (%)	99	96 (97)	278	268 (96)	0.79
Maternal hypertension, *n* (%)	99	25 (25)	279	58 (21)	0.36
Maternal diabetes, *n* (%)	99	6 (6)	279	23 (8)	0.48
Chorioamnionitis, *n* (%)	99	21 (21)	279	40 (14)	0.11
Histologic chorioamnionitis, *n* (%)	93	56 (60)	268	153 (57)	0.60
Rupture of membranes >18 h, *n* (%)	99	26 (26)	274	63 (23)	0.51
Cesarean section, *n* (%)	99	63 (64)	279	177 (63)	0.97
Antenatal steroids, *n* (%)	99	90 (91)	279	261 (94)	0.38
Complete course of antenatal steroids, *n* (%)	99	56 (57)	278	182 (65)	0.11
Singleton, *n* (%)	99	66 (67)	279	193 (69)	0.64

^a^Differences in categorical variables were tested for by Fisher’s exact test or chi-square test; differences in continuous variables by Wilcoxon rank-sum test.

**Table 2:** Neonatal Morbidity and Patent Ductus Arteriosus Treatment Characteristics.

Characteristics	*n*	Catheter closure *n* = 99	*n*	Surgical ligation *n* = 279	*P*-value^a^
Age at PDA procedural closure, days, Median (IQR)	97	62 (42–78)	254	31.5 (22–42)	<0.01
Postmenstrual age at intervention, weeks, Median (IQR)	97	33.9 (31.1–36.6)	254	29.1 (27.7–31.0)	<0.01
Indomethacin	99	29 (29)	277	112 (40)	0.05
Ibuprofen	99	38 (38)	279	94 (34)	0.40
Acetaminophen	99	12 (12)	279	91 (33)	<0.01
Indomethacin within first 24 h	98	8 (8)	278	79 (28)	<0.01
Surfactant	99	98 (99)	279	271 (97)	0.30
Steroid use for BPD	98	49 (50)	270	137 (51)	0.90
Seizures	99	5 (5)	279	8 (3)	0.31
Intraventricular hemorrhage	99	47 (47)	279	109 (39)	0.14
Ventricular enlargement with blood in ventricles or blood in parenchyma	99	28 (28)	279	58 (21)	0.13
PVL	99	16 (16)	279	23 (8)	0.03
Ventricular enlargement after 28 days	95	19 (20)	262	41 (16)	0.33
Cystic PVL/porencephalic cyst after 28 days	95	12 (13)	262	18 (7)	0.08
Early onset sepsis	99	2 (2)	279	11 (4)	0.37
Late onset sepsis	99	33 (33)	279	94 (34)	0.95
Necrotizing enterocolitis	99	16 (16)	278	28 (10)	0.11
Death before discharge	99	2 (2)	275	15 (5)	0.16
ROP, any	99	75 (76)	272	242 (89)	<0.01
Stage 3 or higher	99	24 (24)	272	91 (33)	0.09
Intervention-laser or bevacizumab	96	19 (20)	261	54 (21)	0.85
BPD, any	99	93 (94)	269	248 (92)	0.57
BPD Grade 2	99	41 (41)	269	122 (45)	0.50
BPD Grade 3	99	29 (29)	269	54 (20)	0.06

^a^Differences in categorical variables were tested for by Fisher’s exact test or chi-square test; differences in continuous variables by Wilcoxon rank-sum test.

The primary outcome of death or severe NDI occurred in 49% of infants in TCPC group and 40% in surgical ligation group. Death before neurodevelopmental follow-up occurred in 3% of TCPC group compared to 6% in surgical ligation group but this difference was not statistically significant. Severe NDI among survivors was higher in the TCPC group compared to surgical ligation (47% vs 34%, *p* = 0.04) (Table 3).

**Table 3:** PDA treatment modality and neurodevelopmental and behavioral outcomes at 2 years of age.

Characteristics	*n*	Catheter closure *n* = 99	*n*	Surgical ligation *n* = 279	*P*-value^a^
Death/Severe NDI, *n* (%)	81	40 (49)	215	85 (40)	0.13
Severe NDI, *n* (%)	78	37 (47)	197	67 (34)	0.04
Death by follow-up, *n* (%)	99	3 (3)	279	18 (6)	0.20
Moderate to severe cerebral palsy, *n* (%)	81	13 (16)	211	22 (10)	0.19
BSID Cognitive Composite Score <70, *n* (%)	80	31 (39)	202	45 (22)	<0.01
BSID Language Composite Score <70, *n* (%)	79	36 (46)	196	64 (33)	0.04
BSID Motor Composite Score <70, *n* (%)	77	31 (40)	199	56 (28)	0.05
Blindness, *n* (%)	81	2 (2)	211	2 (1)	0.32
Hearing impairment, *n* (%)	80	3 (4)	203	5 (2)	0.56
Head circumference Z-score at follow-up, Median (IQR)	81	−0.9 (−1.6 to 0.3)	206	−0.9 (−1.7 to −0.1)	0.10
NDI, *n* (%)	79	63 (80)	201	132 (66)	0.02
BSID-III Cognitive Composite Score <85, *n* (%)	80	58 (73)	202	98 (49)	<0.01
BSID-III Language Composite Score <85, *n* (%)	79	62 (78)	196	120 (61)	0.01
BSID-III Motor Composite Score <85, *n* (%)	77	50 (65)	199	112 (56)	0.19

^a^Differences in categorical variables were tested for by Fisher’s exact test or chi-square test; differences in continuous variables by Wilcoxon rank-sum test.

In the adjusted analysis, there was no difference in odds of death or severe NDI between the transcatheter closure and surgical ligation groups (aOR 1.12 [95% CI: 0.55–2.26]) (Table 4). Similarly, there was no difference in odds of death before follow up (aOR 1.41 [95% CI: 0.67–2.96]) or severe NDI (aOR 0.19 [95% CI: 0.03–1.11]) between groups.

**Table 4:** Multivariable logistic regression analysis of death and/or severe neurodevelopmental impairment between transcatheter closure and surgical ligation.

Outcome	Unadjusted Odds Ratio (95% CI)	Adjusted Odds Ratio (95% CI)^a^
Death by follow-up or severe neurodevelopmental impairment	1.49 (0.89, 2.50)	1.12 (0.55, 2.26)
Severe neurodevelopmental impairment	1.75 (1.03, 2.98)	1.41 (0.67, 2.96)
Death by follow-up	0.36 (0.08, 1.59)	0.19 (0.03, 1.11)

^a^Logistic regression models adjusted for center, birth year, gestational age, birth weight, and age at treatment.

## Discussion

This multicenter retrospective cohort study showed that the odds of severe NDI or death were similar among extremely preterm infants undergoing TCPC and surgical ligation. This is the first large multicenter study to evaluate 2-year neurodevelopmental outcomes in preterm infants undergoing TCPC.

Our results are consistent with the findings from two studies comparing neurodevelopmental outcomes of infants undergoing TCPC compared to surgical ligation [16, 17]. It should be noted that these comparison studies were single-center studies with small sample sizes and grossly underpowered. The study by Fernandez and colleagues [16] reported similar timing of PDA procedural closure to the current study, where surgical ligation was performed at an earlier age compared to TCPC.

Patients undergoing surgical PDA ligation compared to patients undergoing TCPC have been shown to be more likely to require longer duration of general anesthesia, have increased need for pain and sedation medications, have longer duration of mechanical ventilation and have increased risk for post-ligation cardiac syndrome and recurrent laryngeal nerve palsy [21]. Theoretically, the increased likelihood of adverse effects after surgical ligation may place this population at increased risk of developing neurodevelopmental impairment. Evidence suggests that suboptimal cerebral oxygenation due to prolonged PDA shunting may affect brain growth leading to adverse neurodevelopmental outcomes [22, 23]. Of note, infants in the TCPC group had higher incidence of PVL (16% vs 8%) and higher incidence of severe NDI (47% vs 34%) compared to the surgical ligation group. However, the TCPC group had much later median age of intervention and so presumably had a greater duration of PDA shunt exposure. Whether earlier TCPC resulting in shorter duration of altered cerebral oxygenation could have led to improved neurodevelopmental outcomes is not known.

Since the FDA approval of the Amplatzer Piccolo Occluder in 2019, a device for TCPC in infants as small as 700 g, TCPC has been increasingly utilized as a strategy to achieve definitive closure of the PDA [1–3]. In more recent years, TCPC has surpassed surgical ligation as the most common method of procedural closure [1–3, 9, 10]. The safety profile of TCPC has improved over time with increasing case volume and expertise [24]. Along with the rapid adoption of TCPC, the age and size of infants undergoing TCPC have declined over time [2, 3]. It should be noted that this study includes subjects born only until 12/31/2019 indicating that likely a small percentage of infants could have gotten the Amplatzer Piccolo Occluder as centers were adapting the use of this device into clinical practice.

Beyond how TCPC compares to surgical ligation, it is important for neonatologists and interventional cardiologists to understand how TCPC compares to conservative management of hemodynamically significant PDA. This question is currently being addressed by the ongoing clinical trial “Percutaneous Intervention Versus Observational Trial of Arterial Ductus in Low Weight Infants (PIVOTAL).” [25] PIVOTAL is assessing neurodevelopmental outcomes at 36 weeks’ postmenstrual age and 3-4 months’ corrected age as secondary outcomes.

Strengths of this study include its large sample size compared to previously published studies. Data were prospectively collected by trained research staff which is another important strength of the study. However, our results have several important limitations. *First*, there was no standardized echocardiography protocol across NRN sites nor a standardized definition of a hemodynamically significant PDA. *Second*, neither the timing of diagnosis of PDA nor the burden (severity or duration) of shunt exposure was available. *Third*, the study cohort included infants born through the end of 2019 and so describe several years prior to widespread adoption of the Amplatzer Piccolo Occluder into clinical practice. *Fourth*, confounding by indication and residual confounding by factors not accounted for in our models (eg., PDA-targeted medication exposure, severity of illness contributing to patency of the PDA) could have biased our results. *Fifth*, occurrence of post-ligation cardiac syndrome (or post-transcatheter cardiorespiratory syndrome) and development of chronic pulmonary hypertension, both of which could be associated with adverse neurodevelopmental outcomes [21, 26], were not recorded in our dataset.

## Conclusion

These data provide the first large multisite comparison of TCPC and surgical ligation on 2-year follow-up outcomes including neurodevelopmental impairment. As the age at TCPC declines, our findings should be re-evaluated in prospective studies that include infants receiving non-procedural treatment of the PDA as the comparison group and with better assessment of the magnitude and duration of PDA shunt physiology and related hemodynamic factors.

### Data Sharing

Data reported in this paper may be requested through a data use agreement. Further details are available at https://neonatal.rti.org/index.cfm?fuseaction=DataRequest.Home.

## Data Availability

Data reported in this paper may be requested through a data use agreement. Further details are available at https://neonatal.rti.org/index.cfm?fuseaction=DataRequest.Home.
